# Two cases of perivascular epithelioid cell tumor of the uterus: clinical, radiological and pathological diagnostic challenge

**DOI:** 10.1186/s40001-017-0248-y

**Published:** 2017-03-07

**Authors:** Byung Su Kwon, Dong Soo Suh, Nam Kyung Lee, Yong Jung Song, Kyung Un Choi, Ki Hyung Kim

**Affiliations:** 1 Department of Obstetrics and Gynecology, Pusan National University School of Medicine, 179, Gudeok-Ro, Seo-Gu, Busan, 49241 South Korea; 2 Biomedical Research Institute and Pusan Cancer Center, Pusan National University Hospital, 179, Gudeok-Ro, Seo-Gu, Busan, 49241 South Korea; 3 Department of Radiology, Pusan National University School of Medicine, Busan, 49241 South Korea; 4 Department of Pathology, Pusan National University School of Medicine, Busan, 49241 South Korea

**Keywords:** Perivascular epithelioid cell tumor (PEComa), Morphology, Immunohistochemistry

## Abstract

**Background:**

Perivascular epithelioid cell tumor (PEComa) is a rare subtype of mesenchymal origin tumor composed of epithelioid cells which exhibits immunohistochemical co-expressions of melanocytic markers and smooth muscle markers.

**Case presentation:**

In the first case, malignant uterine PEComa with vaginal and multiple lung metastasis was misdiagnosed preoperatively as uterine leiomyosarcoma despite a preoperative punch biopsy and immunohistochemical analysis of the metastatic vaginal mass. In the second case, synchronous uterine PEComa showing benign histology with lymph node involvement was incidentally detected after a staging operation for ovarian cancer. Definitive diagnosis of uterine PEComa was achieved only after hysterectomy despite preoperative assessment with pelvic magnetic resonance imaging and punch biopsy of metastatic lesion.

**Conclusion:**

The authors report two rare cases of uterine PEComa diagnosed postoperatively based on the morphologic and immunohistochemical features.

## Background

Perivascular epithelioid cell tumor (PEComa) is a rare subtype of mesenchymal origin tumors, and is composed of perivascular epithelioid cells with specific histologic and immunohistochemical features [1]. PEComa of the female gynecological tract is a rare entity presenting with variable symptoms and different prognosis for each individual case. The uterus is one of the most commonly involved sites. Preoperative distinction may at times be problematic, as the symptoms of uterine PEComa are nonspecific and similar to those of other uterine tumors, and some patients may be asymptomatic.

Radiologically, uterine PEComas can be highly homogeneous, resembling small, benign smooth muscle lesions, and thus, their differential diagnosis is almost impossible based on imaging features alone [2]. However, their preoperative diagnosis may be assisted by biopsy and subsequent immunohistochemistry, which is nearly always immunoreactive for both melanocytic (HMB-45, melan-A, MiTF) and smooth muscle markers (SMA, desmin, caldesmon) [3]. Herein, the authors present two rare cases of uterine PEComa diagnosed postoperatively.

## Case presentation

### Case 1

**Fig. 1:**
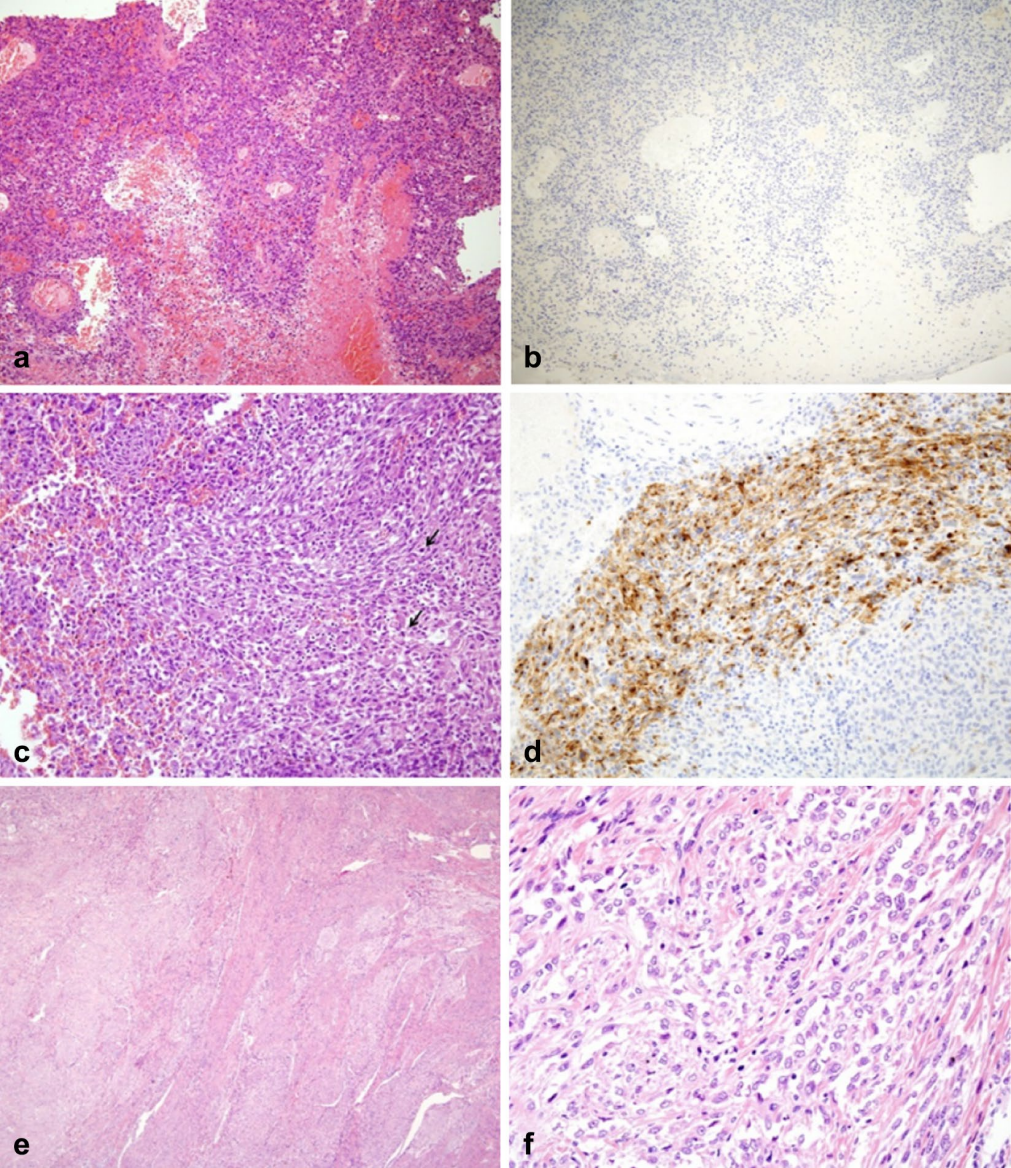
Microscopic findings of malignant uterine PEComa (case 1, **a**–**d**). Punch biopsy from vagina (**a**, **b**); section showing atypical epithelioid tumor cells with marked necrosis (**a**, original magnification ×100). Immunostaining was negative for HMB45 (**b**, ×100). Tumor mass obtained by hysterectomy (**c**, **d**); section revealing epithelioid tumor cells with cytologic atypia and high mitotic activity (arrow) (**c**, ×200). The tumor cells showed patchy HMB45 expression by immunostaining (**d**, ×200). Microscopic findings of uterine mass (case 2, **e**, **f**). The tumor showed local infiltration into adjacent uterine smooth muscle (**e**, original magnification ×40) and predominantly benign-looking epithelioid tumor cells (**f**, ×400)

**Fig. 2:**
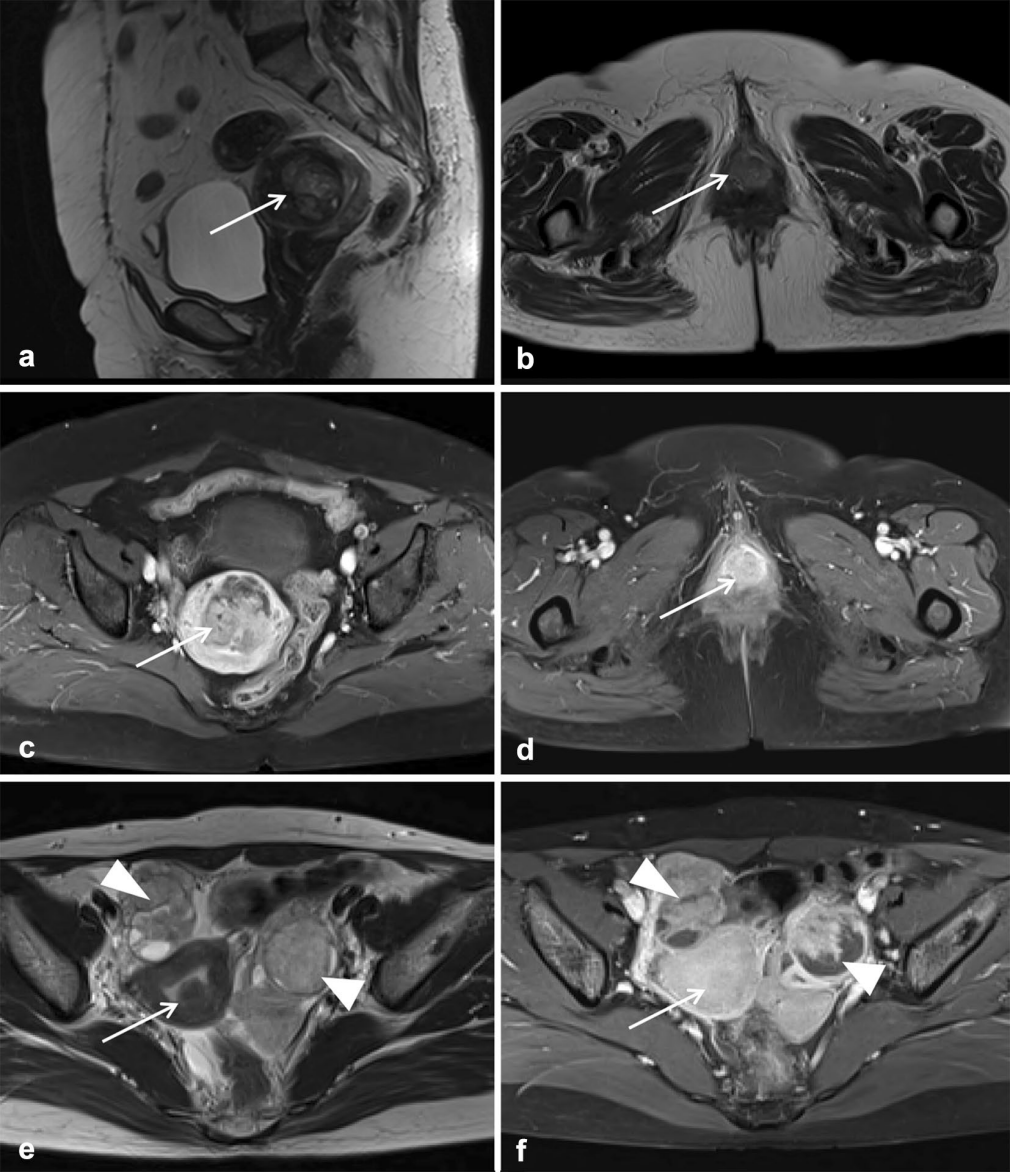
Preoperative pelvic MRI findings of malignant uterine PEComa (case 1, **a**–**d**). Sagittal T2-weighted (**a**) and axial T2-weighted (**b**) images showed two masses with heterogeneous hyperintensity in the uterine myometrium (*arrow* in **a**) and the lower vagina (*arrow* in **b**). Axial fat-suppressed contrast-enhanced T1-weighted images (**c**, **d**) showed the enhancement of the masses (*arrows* in **c**, **d**). Preoperative pelvic MRI findings of benign uterine PEComa (case 2, **e**, **f**). Axial T2-weighted (**e**) and fat-suppressed contrast-enhanced T1-weighted (**f**) images showed a well-defined, homogeneous submucosal uterine mass (*arrows*), showing signal intensity or enhancement similar to that of the myometrium. Note bilateral ovarian complex cystic masses with solid enhancing portions (*arrowheads*)

Patient 1 is a 62-year-old woman who presented with intermittent vaginal bleeding for 2 weeks. Her past medical history was unremarkable. Initial findings were a heterogenous longitudinal uterine mass of length 4.6 cm on ultrasonography and a hard, whitish, 2.0-cm sized mass arising from anterior vagina. A punch biopsy of vaginal mass was performed. Histologic and immunohistochemical findings (positive for SMA, but negative for HMB45) were malignant tumor, suggestive of leiomyosarcoma (Fig. 1a, b). Pelvic magnetic resonance imaging (MRI) revealed a 4.6 × 5.5 cm sized heterogeneous enhancing mass in the uterine myometrium (Fig. 2a, b). Additionally, a 2.1 × 1.1 cm sized indistinct, heterogeneous, enhancing mass was observed in the lower vagina (Fig. 2c, d). Subsequent chest computerized tomography (CT) revealed multiple pulmonary metastatic nodules. Consequently, the patient underwent explorative laparotomy and surgical staging operation (Table 1). Histologic examination revealed that the tumor cells were predominantly composed of epithelioid cells with eosinophilic cytoplasm and showed cytologic atypia, high mitotic activity (4/10 HPFs), an infiltrative growth pattern, and necrosis. Immunohistochemistry showed patchy expressions of HMB45 and SMA. Tumor cells were negative for PAN-CK, desmin, S-100, melan-A, EMA, and CD10 (Fig. 1c, d). In combination of these pathological features and the Immunohistochemistry results, the final pathologic diagnosis was of malignant perivascular epithelioid cell tumor (PEComa). The mass from vagina displayed the same histologic findings. The patient was started on the mTOR inhibitor everolimus at 25 mg i.v. weekly. She is undergoing surveillance at 9 months of follow-up.

**Table 1:** Summary of clinicopathologic characteristics of the two cases of uterine PEComa

Characteristics	Case 1 (uterine PEComa, malignant)	Case 2 (uterine PEComa, benign)
Age (year)	62	38
Association of TSC	No	Yes
Synchronous cancer	No	Yes (epithelial ovarian cancer)
Initial presentation	Abnormal uterine bleeding	No
Tumor site/size (cm)	Myometrial mass in uterus/4.6 cm; anterior mass in vaginal wall/1.2 cm	Submucosal mass in uterus/4.6 cm
Extent of disease	Involvement of both lung as well as anterior vagina	Involvement of peri-aortic lymph nodes
Radiologic findings (MRI)	Degenerative fibroid or leiomyosarcoma	Leiomyoma
Histologic findings	Epithelioid; infiltrative; tumor necrosis present; cytologic atypia present; high mitotic activity (4/10HPFs)	Epithelioid; focal Infiltrative; major cytological or nuclear pleomorphism absent; low mitotic rate;
Positive IHC profile	HMB-45, SMA	HMB-45, SMA
Negative IHC profile	PAN-CK, desmin, S-100, melan-A, EMA, CD10	PAN-CK, desmin, S-100, melan-A, EMA, CD10, c-kit
Treatment		
Surgical	Hysterectomy; BSO; partial omentectomy; appendectomy; mass resection in the anterior vagina	Hysterectomy; BSO; infracolic omentectomy; pelvic LN dissection; para-aortic LN dissection; appendectomy
Adjuvant	mTOR inhibitor—everolimus (weekly, ongoing)	Paclitaxel and carboplatin CTX (6 cycles of tri-weekly)
Follow-up	Lung and bone metastases, AWD at 18 months	ANED at 6 months

*PEComas* perivascular epithelioid tumors, *TSC* tuberous sclerosis complex, *MRI* magnetic resonance imaging, *HPF* high power field, *IHC* immunohistochemical, *HMB-45* human melanoma black 45, *SMA* smooth muscle actin, *PAN-CK* pan-cytokeratin, *EMA* epithelial membrane antigen, *BSO* bilateral salpingo-oophorectomy, *CTX* chemotherapy, *AWD* alive with disease, *ANED* alive with no evidence of disease

### Case 2

Patient 2 is a 38-year-old woman who presented with an adnexal mass and highly elevated CA125 level (3979 U/mL). The patient had history of tuberous sclerosis. Pelvic MRI revealed complex cystic masses with enhancing solid portions in both ovaries, suggestive of primary ovarian malignancy. MRI also showed a 1.5 × 1.0-cm sized well-circumscribed, homogenous, submucosal uterine mass showing signal intensity or enhancement similar to that of the myometrium, suggestive of uterine leiomyoma (Fig. 2e, f). Optimal cytoreductive surgery was performed. Histologic examination revealed that serous papillary carcinoma of the ovary with multiple peritoneal metastases and intrauterine mass consisted of predominantly epithelioid tumor cells and focal infiltrative growth into myometrium. Epithelioid tumor cells showed no major cytological or nuclear pleomorphism and low mitotic activity (Fig. 1e, f). Sections from peri-aortic lymph nodes showed involvement of PEComa. Immunohistochemistry revealed that tumor cells were positive for HMB45 and SMA, but negative for PAN-CK, desmin, S-100, melan-A, EMA, c-kit, and CD10. These morphologic features and the immunohistochemical staining pattern confirmed a diagnosis of PEComa showing benign histology with involvement of peri-aortic lymph nodes. After surgery, the patient completed six cycles of adjuvant chemotherapy (paclitaxel and carboplatin) for ovarian cancer and was followed up regularly. She had no evidence of disease 8 months after treatment completion.

These morphologic features and the immunohistochemical staining pattern confirmed a diagnosis of PEComa.

## Discussion

Perivascular epithelioid cell tumor (PEComa) is recently described entity in the gynecological tract. It occurs most commonly in the retroperitoneum, abdominopelvic region, and uterus. To date, around 78 case reports have been issued on uterine PEComas and the most com-mon site in the female genital tract. We present two cases of PEComa of the uterus with different clinicopathological features (Table 1).

Clinical, radiological, and preoperative pathological features of PEComa cases are diagnostic challenge. In most of the PEComa cases, the diagnosis was confirmed after hysterectomy for a presumed benign disease. The proper preoperative diagnosis of uterine PEComa is difficult because of the lack of a reliable method to distinguish uterine PEComa from benign leiomyomas and uterine sarcoma preoperatively in addition to the rarity of such cases. Clinical manifestations are not useful to distinguish between uterine PEComas and other uterine tumors, since most of the patients with uterine PEComas show abnormal uterine bleeding or lower abdominal pain with a palpable mass, which is similar to the symptoms and signs of other uterine tumors. PEComa can be associated with tuberous sclerosis complex (TSC). Some PEComas occur in TSC patients in sites, such as, kidney, liver, and lung. However, uterine PEComa associated with tuberous sclerosis is very rare.

Imaging features of PEComas are nonspecific and mimic other benign and malignant neoplasms. One study by Tan et al. [4] reported the largest imaging series of 36 patients with malignant PEComas. Ultrasound showed a heterogeneous echotexture with no cystic areas or significant vascularity on Doppler examination. By a comparison with skeletal muscle, CT scans revealed a rather well-defined hypo- or isointense structure, and MRI also displayed a hypo- to isointense structure on T1-weighted imaging and a heterogeneously hyperintense structure on T2-weighted imaging. In general, the characteristics of contrast-enhanced CT or MRI of malignant PEComas were very variable, but their results showed significant enhancement on CT and MRI [2]. Despite such radiologic features, the differential diagnosis of these diseases from other uterine tumors is almost impossible based on imaging features alone. In our two cases, we performed imaging with MRI but failed to distinguish between uterine PEComas and other uterine tumors. One of these two tumors was diagnosed as a degenerative fibroid or leiomyosarcoma because of the large tumor size and the presence of necrotic areas. The other was diagnosed as leiomyoma because of the homogeneous enhancement (Fig. 2a, b). MRI did not provide a definitive diagnosis in our cases.

Since PEComas are nearly always immunoreactive for both melanocytic (HMB-45, melan-A, MiTF) and smooth muscle (actin, desmin, caldesmon) markers, characteristic histologic and immunohistochemical findings provide the most accurate means of diagnosis [3]. Preoperatively, biopsy is not usually performed in uterine tumors, and it is very limited in many cases. In our case 1, we failed to differentiate uterine PEComa and leiomyosarcoma despite a preoperative punch biopsy and immunohistochemical analysis of the metastatic vaginal mass. Preoperative diagnosis was malignant tumor suggestive of leiomyosarcoma, mainly based on the immunohistochemical results (positive for SMA, but negative for HMB45). However, the final immunohistochemical result showed patchy expression for HMB45. Therefore, it was finally diagnosed as malignant PEComa in conjunction with histologic and immunohistochemical findings. To prevent diagnostic error, multiple pieces of biopsy should be sampled in case of metastatic disease. However, morphology- and immunohistochemistry-based diagnosis may lead to incorrect or inconclusive diagnoses due to patchy expression of HMB45 in tumor cells and a marked necrotic background [5].

Although uterine PEComas represent a spectrum of histologies ranging from benign to malignant lesions, the tumor aggressiveness has not yet been fully explained. Malignant uterine PEComas can spread to the vagina, fallopian tubes, ovaries, bladder, and ureters. Distant metastasis to the lungs or liver has also been reported. Up to 20% of women with malignant uterine PEComa have an additional uterine disease at presentation [2]. Folpe et al. reported that PEComas are divided into benign, uncertain malignant potential (UMP), or malignant tumors according to the following six risk factors: tumor size ≥5 cm, infiltrative growth pattern, high nuclear grade cellularity, mitotic rate >1/50 high power fields (HPF), necrosis or vascular invasion [3]. Schoolmeister et al. revised the classification of PEComas. The tumor is considered as malignant when there are more than four unfavorable prognostic indicators (size ≥5 cm, mitoses ≥1/50 HPF, significant nuclear atypia, necrosis, and lymphovascular invasion) [6]. Bleeker et al. suggested revised risk stratification criteria utilizing only size ≥5 cm and high mitotic rate, which were the only high-risk histopathologic features significantly associated with recurrence [7].

The most adequate management of gynecological PEComas has not been well established owing to the paucity of the cases. Surgical resection remains the treatment of choice. The vast majority of patients received a total hysterectomy with or without bilateral salpingo-oophorectomy. Radical hysterectomy with bilateral salpingo-oophorectomy should be considered in patients with PEComas spreading or localized in the uterine cervix [8].

## Conclusion

In conclusion, we describe two rare cases of uterine PEComa with different clinicopathological features. Definitive diagnosis was achieved only after hysterectomy by immunohistochemical analysis. Careful analysis of morphologic and immunohistochemical features is necessary for an adequate differential diagnosis from other mesenchymal tumors. Based on our experience of these cases, PEComa should be considered when a uterine tumor is detected despite its rarity.

## Abbreviations

PEComa: perivascular epithelioid cell tumor
UMP: uncertain malignant potential
MRI: magnetic resonance imaging
CT: computerized tomography
CA-125: carbohydrate antigen-125
HPF: high power fields
